# Diaqua­bis­(2-iodo­benzoato-κ*O*)bis­(nicotinamide-κ*N* ^1^)copper(II)

**DOI:** 10.1107/S1600536812034587

**Published:** 2012-08-08

**Authors:** Ömür Aydın, Nagihan Çaylak Delibaş, Hacali Necefoğlu, Tuncer Hökelek

**Affiliations:** a Department of Chemistry, Kafkas University, 36100 Kars, Turkey; b Department of Physics, Sakarya University, 54187 Esentepe, Sakarya, Turkey; c Department of Physics, Hacettepe University, 06800 Beytepe, Ankara, Turkey

## Abstract

In the title complex, [Cu(C_7_H_4_IO_2_)_2_(C_6_H_6_N_2_O)_2_(H_2_O)_2_], the Cu^II^ cation is located on an inversion center and is coordinated by two monodentate 2-iodo­benzoate (IB) anions, two nicotinamide (NA) ligands and two water mol­ecules in a distorted octa­hedral coordination geometry. The dihedral angle between the carboxyl­ate group and the adjacent benzene ring is 32.12 (14)°, while the pyridine ring and the benzene ring are oriented at a dihedral angle of 82.02 (5)°. The coordinating water mol­ecule links with the carboxyl­ate group *via* an intra­molecular O—H⋯O hydrogen bond. In the crystal, N—H⋯O, O—H⋯O and weak C—H⋯O hydrogen bonds link the mol­ecules into a three-dimensional supra­molecular network.

## Related literature

For literature on niacin, see: Krishnamachari (1974 ▶). For information on the nicotinic acid derivative *N*,*N*-diethyl­nicotinamide, see: Bigoli *et al.* (1972 ▶). For related structures, see: Aydın *et al.* (2012 ▶); Hökelek *et al.* (2009 ▶); Necefoğlu *et al.* (2011 ▶); Sertçelik *et al.* (2012**a* ▶,b* ▶); Sertçelik *et al.* (2009 ▶). For bond-length data, see: Allen *et al.* (1987 ▶).
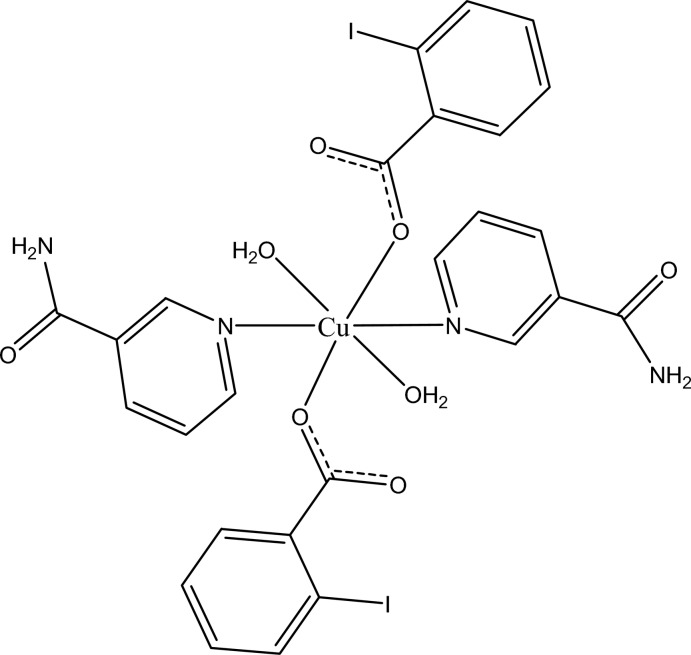


## Experimental

### 

#### Crystal data

[Cu(C_7_H_4_IO_2_)_2_(C_6_H_6_N_2_O)_2_(H_2_O)_2_]
*M* *_r_* = 837.85
Monoclinic, 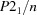

*a* = 8.1617 (2) Å
*b* = 18.3365 (4) Å
*c* = 9.7047 (3) Å
β = 103.573 (3)°
*V* = 1411.81 (7) Å^3^
*Z* = 2
Mo *K*α radiation
μ = 3.02 mm^−1^
*T* = 100 K
0.39 × 0.36 × 0.24 mm

#### Data collection

Bruker Kappa APEXII CCD area-detector diffractometer
Absorption correction: multi-scan (*SADABS*; Bruker, 2005 ▶) *T*_min_ = 0.528, *T*_max_ = 0.661
13216 measured reflections
3531 independent reflections
3337 reflections with *I* > 2σ(*I*)
*R*_int_ = 0.018

#### Refinement

*R*[*F* ^2^ > 2σ(*F* ^2^)] = 0.021
*wR*(*F* ^2^) = 0.058
*S* = 1.13
3531 reflections
203 parameters
H atoms treated by a mixture of independent and constrained refinement
Δρ_max_ = 0.97 e Å^−3^
Δρ_min_ = −0.50 e Å^−3^

### 

Data collection: *APEX2* (Bruker, 2007 ▶); cell refinement: *SAINT* (Bruker, 2007 ▶); data reduction: *SAINT*; program(s) used to solve structure: *SHELXS97* (Sheldrick, 2008 ▶); program(s) used to refine structure: *SHELXL97* (Sheldrick, 2008 ▶); molecular graphics: *ORTEP-3 for Windows* (Farrugia, 1997 ▶); software used to prepare material for publication: *WinGX* (Farrugia, 1999 ▶) and *PLATON* (Spek, 2009 ▶).

## Supplementary Material

Crystal structure: contains datablock(s) I, global. DOI: 10.1107/S1600536812034587/xu5599sup1.cif

Structure factors: contains datablock(s) I. DOI: 10.1107/S1600536812034587/xu5599Isup2.hkl

Additional supplementary materials:  crystallographic information; 3D view; checkCIF report

## Figures and Tables

**Table 1:** Selected bond lengths (Å)

Cu1—O1	1.9937 (14)
Cu1—O4	2.5078 (16)
Cu1—N1	1.9984 (16)

**Table 2:** Hydrogen-bond geometry (Å, °)

*D*—H⋯*A*	*D*—H	H⋯*A*	*D*⋯*A*	*D*—H⋯*A*
N2—H21⋯O3^i^	0.84 (3)	2.17 (3)	2.942 (3)	154 (3)
N2—H22⋯O2^ii^	0.83 (3)	2.11 (3)	2.881 (2)	154 (3)
O4—H41⋯O2^iii^	0.87 (4)	1.87 (4)	2.720 (2)	165 (4)
O4—H42⋯O3^iv^	0.80 (4)	2.16 (4)	2.923 (2)	160 (4)
C10—H10⋯O2^ii^	0.93	2.49	3.368 (2)	158

Symmetry codes: (i) 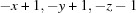; (ii) 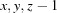; (iii) 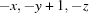; (iv) 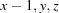.

**Fig. 1.**
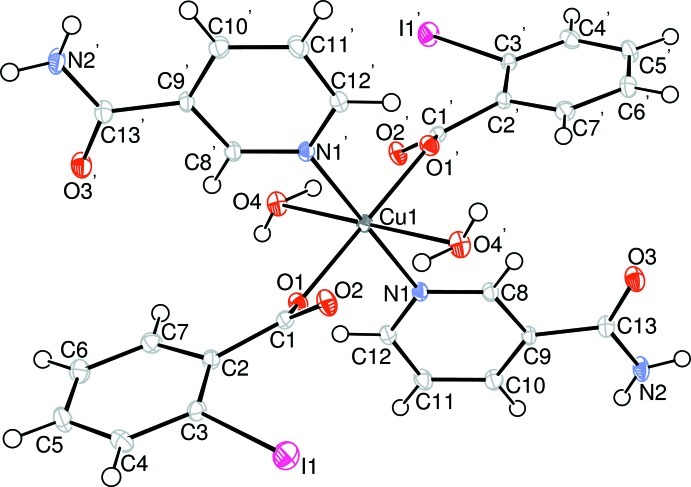
The molecular structure of the title molecule with the atom-numbering scheme. Displacement ellipsoids are drawn at the 50% probability level [symmetry code: (') -x, 1-y, -z].

## supplementary crystallographic information

### Comment

As a part of our ongoing investigations of transition metal complexes of nicotinamide (NA), one form of niacin (Krishnamachari, 1974), and/or the nicotinic acid derivative *N*,*N*-diethylnicotinamide (DENA), an important respiratory stimulant (Bigoli *et al.*, 1972), the title compound was synthesized and its crystal structure is reported herein.

In the title mononuclear complex, Cu^II^ cation is located on an inversion center and is coordinated by two 2-iodobenzoate (IB) anions, two nicotinamide (NA) ligands and two water molecules, all ligands coordinating in a monodentate manner (Fig. 1). The crystal structures of similar complexes of Cu^II^, Co^II^, Ni^II^, Mn^II^ and Zn^II^ ions, [Cu(C_7_H_4_BrO_2_)_2_(C_6_H_6_N_2_O)_2_(H_2_O)_2_] (Necefoğlu *et al.*, 2011), [Co(C_7_H_4_IO_2_)_2_(C_6_H_6_N_2_O)_2_(H_2_O)_2_] (Aydın *et al.*, 2012), [Ni(C_8_H_5_O_3_)_2_(C_6_H_6_N_2_O)_2_(H_2_O)_2_] (Sertçelik *et al.*, 2012*a*), [Mn(C_8_H_5_O_3_)_2_(C_10_H_14_N_2_O)_2_(H_2_O)_2_] (Sertçelik *et al.*, 2009), [Zn(C_7_H_4_BrO_2_)_2_(C_6_H_6_N_2_O)_2_(H_2_O)_2_] (Hökelek *et al.*, 2009) and [Zn(C_8_H_5_O_3_)_2_(C_6_H_6_N_2_O)_2_(H_2_O)_2_] (Sertçelik *et al.*, 2012*b*) have also been reported, where all the ligands coordinate to the metal atoms in a monodentate manner.

In the title complex, the four symmetry related O atoms (O1, O1', O4 and O4') in the equatorial plane around the Cu^II^ ion form a slightly distorted square-planar arrangement, while the slightly distorted octahedral coordination is completed by the two symmetry related N atoms of the NA ligands (N1 and N1') in the axial positions. The near equalities of the C1—O1 [1.275 (2) Å] and C1—O2 [1.245 (2) Å] bonds in the carboxylate group indicate delocalized bonding arrangement, rather than localized single and double bonds. The Cu—O bond lengths are 1.9937 (14) Å (for benzoate oxygens) and 2.5078 (16) Å (for water oxygens), and the Cu—N bond length is 1.9984 (16) Å, close to standard values (Allen *et al.*, 1987). The Cu atom is displaced out of the mean-plane of the carboxylate group (O1/C1/O2) by -0.5995 (1) Å. The dihedral angle between the planar carboxylate group and the adjacent benzene ring A (C2—C7) is 32.12 (14)°. The benzene A (C2—C7) and the pyridine B (N1/C8—C12) rings are oriented at a dihedral angle of A/B = 82.02 (5)°. The coordinating water molecule links with the carboxylate group *via* an O—H···O hydrogen bond (Table 1).

In the crystal, intermolecular N—H···O, O—H···O and weak C—H···O hydrogen bonds (Table 1) link the molecules into a three-dimensional supramolecular network, in which they may be effective in the stabilization of the structure.

### Experimental

The title compound was prepared by the reaction of CuSO_4_.5H_2_O (1.248 g, 5 mmol) in H_2_O (200 ml) and NA (1.220 g, 200 mmol) in H_2_O (20 ml) with 2-iodobenzoic acid (2.700 g, 10 mmol) in H_2_O (20 ml) at room temperature. The mixture was filtered and set aside to crystallize at ambient temperature for one week, giving blue single crystals.

### Refinement

Atoms H21 and H22 (for NH_2_) and H41 and H42 (for H_2_O) were located in a difference Fourier map and were refined freely. The C-bound H-atoms were positioned geometrically with C—H = 0.93 Å for aromatic H-atoms, and constrained to ride on their parent atoms, with *U*_iso_(H) = 1.2 × *U*_eq_(C).

### Crystal data

**Table:**

[Cu(C_7_H_4_IO_2_)_2_(C_6_H_6_N_2_O)_2_(H_2_O)_2_]	*F*(000) = 814
*M**_r_* = 837.85	*D*_x_ = 1.971 Mg m^−^^3^
Monoclinic, *P*2_1_/*n*	Mo *K*α radiation, λ = 0.71073 Å
Hall symbol: -P 2yn	Cell parameters from 7325 reflections
*a* = 8.1617 (2) Å	θ = 2.8–28.3°
*b* = 18.3365 (4) Å	µ = 3.02 mm^−^^1^
*c* = 9.7047 (3) Å	*T* = 100 K
β = 103.573 (3)°	Block, blue
*V* = 1411.81 (7) Å^3^	0.39 × 0.36 × 0.24 mm
*Z* = 2	

### Data collection

**Table:**

Bruker Kappa APEXII CCD area-detector diffractometer	3531 independent reflections
Radiation source: fine-focus sealed tube	3337 reflections with *I* > 2σ(*I*)
Graphite monochromator	*R*_int_ = 0.018
φ and ω scans	θ_max_ = 28.4°, θ_min_ = 2.2°
Absorption correction: multi-scan (*SADABS*; Bruker, 2005)	*h* = −10→10
*T*_min_ = 0.528, *T*_max_ = 0.661	*k* = −24→24
13216 measured reflections	*l* = −10→12

### Refinement

**Table:**

Refinement on *F*^2^	Primary atom site location: structure-invariant direct methods
Least-squares matrix: full	Secondary atom site location: difference Fourier map
*R*[*F*^2^ > 2σ(*F*^2^)] = 0.021	Hydrogen site location: inferred from neighbouring sites
*wR*(*F*^2^) = 0.058	H atoms treated by a mixture of independent and constrained refinement
*S* = 1.13	*w* = 1/[σ^2^(*F*_o_^2^) + (0.0285*P*)^2^ + 1.437*P*] where *P* = (*F*_o_^2^ + 2*F*_c_^2^)/3
3531 reflections	(Δ/σ)_max_ = 0.001
203 parameters	Δρ_max_ = 0.97 e Å^−^^3^
0 restraints	Δρ_min_ = −0.50 e Å^−^^3^

### Special details

**Table:**

Geometry. All esds (except the esd in the dihedral angle between two l.s. planes) are estimated using the full covariance matrix. The cell esds are taken into account individually in the estimation of esds in distances, angles and torsion angles; correlations between esds in cell parameters are only used when they are defined by crystal symmetry. An approximate (isotropic) treatment of cell esds is used for estimating esds involving l.s. planes.
Refinement. Refinement of F^2^ against ALL reflections. The weighted R-factor wR and goodness of fit S are based on F^2^, conventional R-factors R are based on F, with F set to zero for negative F^2^. The threshold expression of F^2^ > 2sigma(F^2^) is used only for calculating R-factors(gt) etc. and is not relevant to the choice of reflections for refinement. R-factors based on F^2^ are statistically about twice as large as those based on F, and R- factors based on ALL data will be even larger.

### Fractional atomic coordinates and isotropic or equivalent isotropic displacement parameters (Å^2^)

**Table:**

	*x*	*y*	*z*	*U*_iso_*/*U*_eq_	
Cu1	0.0000	0.5000	0.0000	0.01102 (8)	
I1	0.120179 (18)	0.213804 (8)	0.323774 (16)	0.02225 (6)	
O1	−0.10863 (18)	0.40869 (8)	0.04600 (14)	0.0137 (3)	
O2	0.10292 (18)	0.37645 (8)	0.22737 (15)	0.0163 (3)	
O3	0.4441 (2)	0.48850 (9)	−0.32723 (15)	0.0195 (3)	
O4	−0.2967 (2)	0.54420 (9)	−0.08965 (16)	0.0174 (3)	
H41	−0.252 (5)	0.571 (2)	−0.145 (4)	0.045 (10)*	
H42	−0.369 (5)	0.521 (2)	−0.140 (4)	0.056 (12)*	
N1	0.0134 (2)	0.45775 (9)	−0.18686 (17)	0.0116 (3)	
N2	0.3517 (2)	0.42184 (11)	−0.52508 (19)	0.0191 (4)	
H21	0.434 (4)	0.4370 (16)	−0.555 (3)	0.024 (7)*	
H22	0.276 (4)	0.3983 (16)	−0.578 (3)	0.026 (8)*	
C1	−0.0459 (2)	0.37253 (10)	0.1582 (2)	0.0116 (3)	
C2	−0.1668 (2)	0.32473 (11)	0.2144 (2)	0.0117 (3)	
C3	−0.1182 (2)	0.26188 (11)	0.2949 (2)	0.0120 (3)	
C4	−0.2309 (3)	0.22441 (11)	0.3571 (2)	0.0162 (4)	
H4	−0.1958	0.1831	0.4116	0.019*	
C5	−0.3955 (3)	0.24892 (12)	0.3375 (2)	0.0180 (4)	
H5	−0.4703	0.2247	0.3807	0.022*	
C6	−0.4487 (3)	0.30965 (12)	0.2535 (2)	0.0177 (4)	
H6	−0.5599	0.3254	0.2382	0.021*	
C7	−0.3354 (3)	0.34687 (11)	0.1925 (2)	0.0147 (4)	
H7	−0.3722	0.3873	0.1359	0.018*	
C8	0.1566 (2)	0.46254 (10)	−0.2307 (2)	0.0119 (4)	
H8	0.2495	0.4847	−0.1717	0.014*	
C9	0.1720 (2)	0.43569 (10)	−0.36104 (19)	0.0113 (3)	
C10	0.0335 (3)	0.40108 (11)	−0.4471 (2)	0.0153 (4)	
H10	0.0408	0.3815	−0.5338	0.018*	
C11	−0.1159 (3)	0.39604 (12)	−0.4024 (2)	0.0164 (4)	
H11	−0.2101	0.3733	−0.4585	0.020*	
C12	−0.1209 (3)	0.42577 (11)	−0.2720 (2)	0.0142 (4)	
H12	−0.2212	0.4235	−0.2424	0.017*	
C13	0.3339 (3)	0.45019 (11)	−0.4031 (2)	0.0140 (4)	

### Atomic displacement parameters (Å^2^)

**Table:**

	*U*^11^	*U*^22^	*U*^33^	*U*^12^	*U*^13^	*U*^23^
Cu1	0.01365 (16)	0.01161 (15)	0.00929 (15)	−0.00216 (12)	0.00570 (12)	−0.00079 (11)
I1	0.01407 (8)	0.01936 (8)	0.03321 (10)	0.00596 (5)	0.00532 (6)	0.00443 (5)
O1	0.0148 (7)	0.0151 (7)	0.0114 (6)	−0.0026 (5)	0.0036 (5)	0.0004 (5)
O2	0.0112 (7)	0.0219 (7)	0.0152 (7)	−0.0038 (6)	0.0019 (5)	0.0012 (5)
O3	0.0158 (7)	0.0289 (8)	0.0142 (7)	−0.0075 (6)	0.0046 (6)	−0.0058 (6)
O4	0.0148 (7)	0.0222 (8)	0.0150 (7)	−0.0012 (6)	0.0028 (6)	0.0021 (6)
N1	0.0112 (8)	0.0128 (7)	0.0115 (7)	−0.0007 (6)	0.0044 (6)	0.0003 (6)
N2	0.0149 (9)	0.0301 (10)	0.0146 (8)	−0.0072 (8)	0.0082 (7)	−0.0074 (7)
C1	0.0119 (9)	0.0124 (8)	0.0112 (8)	−0.0013 (7)	0.0042 (7)	−0.0025 (6)
C2	0.0115 (9)	0.0128 (8)	0.0115 (8)	−0.0014 (7)	0.0041 (7)	−0.0010 (6)
C3	0.0088 (9)	0.0132 (8)	0.0139 (9)	0.0005 (7)	0.0020 (7)	−0.0011 (7)
C4	0.0185 (10)	0.0138 (9)	0.0163 (9)	−0.0032 (8)	0.0042 (8)	0.0020 (7)
C5	0.0153 (10)	0.0195 (10)	0.0211 (10)	−0.0053 (8)	0.0083 (8)	0.0003 (8)
C6	0.0111 (9)	0.0190 (10)	0.0235 (10)	0.0000 (8)	0.0052 (8)	−0.0006 (8)
C7	0.0142 (9)	0.0136 (9)	0.0160 (9)	0.0006 (7)	0.0030 (7)	0.0008 (7)
C8	0.0113 (9)	0.0135 (9)	0.0113 (8)	−0.0013 (7)	0.0037 (7)	−0.0001 (7)
C9	0.0103 (8)	0.0136 (8)	0.0109 (8)	−0.0011 (7)	0.0044 (7)	0.0002 (6)
C10	0.0148 (10)	0.0195 (10)	0.0121 (9)	−0.0026 (8)	0.0039 (7)	−0.0033 (7)
C11	0.0138 (9)	0.0202 (10)	0.0150 (9)	−0.0058 (8)	0.0028 (7)	−0.0037 (7)
C12	0.0129 (9)	0.0150 (9)	0.0158 (9)	−0.0026 (7)	0.0053 (7)	−0.0008 (7)
C13	0.0111 (9)	0.0183 (9)	0.0128 (9)	−0.0006 (7)	0.0033 (7)	0.0002 (7)

### Geometric parameters (Å, º)

**Table:**

Cu1—O1	1.9937 (14)	C3—C2	1.397 (3)
Cu1—O1^i^	1.9937 (14)	C4—C3	1.394 (3)
Cu1—O4	2.5078 (16)	C4—C5	1.387 (3)
Cu1—O4^i^	2.5078 (16)	C4—H4	0.9300
Cu1—N1	1.9984 (16)	C5—C6	1.388 (3)
Cu1—N1^i^	1.9984 (16)	C5—H5	0.9300
I1—C3	2.0942 (19)	C6—H6	0.9300
O1—C1	1.275 (2)	C7—C6	1.389 (3)
O2—C1	1.245 (2)	C7—H7	0.9300
O3—C13	1.238 (3)	C8—H8	0.9300
O4—H41	0.87 (4)	C9—C8	1.390 (2)
O4—H42	0.80 (4)	C9—C10	1.391 (3)
N1—C8	1.337 (2)	C9—C13	1.496 (3)
N1—C12	1.343 (3)	C10—C11	1.390 (3)
N2—C13	1.331 (3)	C10—H10	0.9300
N2—H22	0.83 (3)	C11—H11	0.9300
N2—H21	0.84 (3)	C12—C11	1.387 (3)
C1—C2	1.513 (3)	C12—H12	0.9300
C2—C7	1.403 (3)		
			
O1^i^—Cu1—O1	180.00 (7)	C4—C5—C6	119.99 (19)
O1—Cu1—N1	89.98 (6)	C4—C5—H5	120.0
O1^i^—Cu1—N1	90.02 (6)	C6—C5—H5	120.0
O1—Cu1—N1^i^	90.02 (6)	C5—C6—C7	119.8 (2)
O1^i^—Cu1—N1^i^	89.98 (6)	C5—C6—H6	120.1
O4—Cu1—O1	84.56 (6)	C7—C6—H6	120.1
O4—Cu1—N1	93.70 (6)	C2—C7—H7	119.3
N1—Cu1—N1^i^	180.00 (9)	C6—C7—C2	121.42 (19)
H41—O4—H42	106 (4)	C6—C7—H7	119.3
C1—O1—Cu1	121.16 (13)	N1—C8—C9	122.62 (18)
C8—N1—Cu1	120.11 (13)	N1—C8—H8	118.7
C12—N1—Cu1	121.13 (13)	C9—C8—H8	118.7
C8—N1—C12	118.74 (16)	C8—C9—C10	118.26 (18)
C13—N2—H21	116 (2)	C8—C9—C13	117.39 (17)
C13—N2—H22	122 (2)	C10—C9—C13	124.23 (17)
H22—N2—H21	120 (3)	C9—C10—H10	120.3
O1—C1—C2	116.35 (17)	C11—C10—C9	119.46 (18)
O2—C1—O1	125.16 (18)	C11—C10—H10	120.3
O2—C1—C2	118.39 (17)	C10—C11—H11	120.8
C3—C2—C1	123.79 (18)	C12—C11—C10	118.33 (19)
C3—C2—C7	117.57 (18)	C12—C11—H11	120.8
C7—C2—C1	118.51 (17)	N1—C12—C11	122.56 (19)
C2—C3—I1	123.71 (14)	N1—C12—H12	118.7
C4—C3—I1	114.92 (15)	C11—C12—H12	118.7
C4—C3—C2	121.30 (18)	O3—C13—N2	122.34 (19)
C3—C4—H4	120.1	O3—C13—C9	120.19 (17)
C5—C4—C3	119.83 (19)	N2—C13—C9	117.45 (18)
C5—C4—H4	120.1		
			
N1—Cu1—O1—C1	123.90 (15)	I1—C3—C2—C7	173.86 (14)
N1^i^—Cu1—O1—C1	−56.10 (15)	C4—C3—C2—C1	172.78 (18)
O1—Cu1—N1—C8	−133.41 (15)	C4—C3—C2—C7	−3.0 (3)
O1^i^—Cu1—N1—C8	46.59 (15)	C5—C4—C3—I1	−176.15 (16)
O1—Cu1—N1—C12	48.21 (16)	C5—C4—C3—C2	1.0 (3)
O1^i^—Cu1—N1—C12	−131.79 (16)	C3—C4—C5—C6	1.5 (3)
Cu1—O1—C1—O2	−20.6 (3)	C4—C5—C6—C7	−1.8 (3)
Cu1—O1—C1—C2	155.73 (13)	C2—C7—C6—C5	−0.4 (3)
Cu1—N1—C8—C9	−178.46 (15)	C10—C9—C8—N1	−1.5 (3)
C12—N1—C8—C9	0.0 (3)	C13—C9—C8—N1	174.79 (18)
Cu1—N1—C12—C11	179.89 (16)	C8—C9—C10—C11	1.6 (3)
C8—N1—C12—C11	1.5 (3)	C13—C9—C10—C11	−174.41 (19)
O1—C1—C2—C3	153.14 (18)	C8—C9—C13—O3	−4.3 (3)
O1—C1—C2—C7	−31.1 (3)	C8—C9—C13—N2	177.18 (19)
O2—C1—C2—C3	−30.3 (3)	C10—C9—C13—O3	171.8 (2)
O2—C1—C2—C7	145.49 (19)	C10—C9—C13—N2	−6.8 (3)
C1—C2—C7—C6	−173.32 (18)	C9—C10—C11—C12	−0.3 (3)
C3—C2—C7—C6	2.7 (3)	N1—C12—C11—C10	−1.3 (3)
I1—C3—C2—C1	−10.3 (3)		

Symmetry code: (i) −*x*, −*y*+1, −*z*.

### Hydrogen-bond geometry (Å, º)

**Table:**

*D*—H···*A*	*D*—H	H···*A*	*D*···*A*	*D*—H···*A*
N2—H21···O3^ii^	0.84 (3)	2.17 (3)	2.942 (3)	154 (3)
N2—H22···O2^iii^	0.83 (3)	2.11 (3)	2.881 (2)	154 (3)
O4—H41···O2^i^	0.87 (4)	1.87 (4)	2.720 (2)	165 (4)
O4—H42···O3^iv^	0.80 (4)	2.16 (4)	2.923 (2)	160 (4)
C10—H10···O2^iii^	0.93	2.49	3.368 (2)	158

Symmetry codes: (i) −*x*, −*y*+1, −*z*; (ii) −*x*+1, −*y*+1, −*z*−1; (iii) *x*, *y*, *z*−1; (iv) *x*−1, *y*, *z*.
